# The legacy of language: What we say, and what people hear, when we talk about genomics

**DOI:** 10.1016/j.xhgg.2023.100231

**Published:** 2023-08-31

**Authors:** Anna Middleton, Alessia Costa, Richard Milne, Christine Patch, Lauren Robarts, Ben Tomlin, Mark Danson, Sasha Henriques, Jerome Atutornu, Ugbaad Aidid, Daniela Boraschi, Catherine Galloway, Keith Yazmir, Sachi Pettit, Tegan Harcourt, Alannah Connolly, Amanda Li, Jacob Cala, Shelby Lake, Julian Borra, Vivienne Parry

**Affiliations:** 1 Wellcome Connecting Science, Wellcome Genome Campus, Hinxton, Cambridgeshire CB10 1SA, UK; 2 Kavli Centre for Ethics, Science and the Public, Faculty of Education, University of Cambridge, 184 Hills Road, Cambridge CB4 8PQ, UK; 3 Maslansky and Partners, 200 Varick Street, Suite 601, New York, NY 10014, USA; 4 The Thin Air Factory Ltd, 71-75 Shelton Street, London WC2H 9JQ, UK; 5 Genomics England, Queen Mary University of London, Dawson Hall, London EC1M 6BQ, UK

**Keywords:** genetics, public, Black, Caribbean, African, Pakistani, engagement, diversity, communication, scientific racism

## Abstract

The way we “talk” about genetics plays a vital role in whether public audiences feel at ease in having conversations about it. Our research explored whether there was any difference between “what we say” and “what people hear” when providing information about genetics to community groups who are known to be missing from genomics datasets. We conducted 16 focus groups with 100 members of the British public who had limited familiarity with genomics and self-identified as belonging to communities with Black African, Black Caribbean, and Pakistani ancestry as well as people of various ancestral heritage who came from disadvantaged socio-economic backgrounds. Participants were presented with spoken messages explaining genomics and their responses to these were analyzed. Results indicated that starting conversations that framed genomics through its potential benefits were met with cynicism and skepticism. Participants cited historical and present injustices as reasons for this as well as mistrust of private companies and the government. Instead, more productive conversations led with an acknowledgment that some people have questions—and valid concerns—about genomics, before introducing any of the details about the science. To diversify genomic datasets, we need to linguistically meet public audiences *where they are at*. Our research has demonstrated that everyday talk about genomics, used by researchers and clinicians alike, is received differently than it is likely intended. We may inadvertently be further disengaging the very audiences that diversity programs aim to reach.

## Teaser

This research demonstrates that there is a difference between “what we say” and “what people hear” when providing information about genetics with community groups who are known to be missing from genomics datasets. Always starting conversations that lead with the benefits of genomics leads to cynicism.

## Introduction

The largest public attitudes survey to document awareness and familiarity with genomics (involving 37,000 people from 22 countries, data gathered in 16 languages) has recently shown that familiarity with genomics and the data-sharing process that underpins research is very low outside of the US.^1^ This work has shown a direct relationship between a lack of awareness of genomics, a mistrust of those using genomic data, and a disinclination of many members of the public to participate in genetics research. It has also shown that those least willing to donate genomic data were people who did not self-identify as white.^2^ More specifically we know that there are community groups in the UK who have explicitly expressed very rational fears of genetics, based on historical injustices and perceptions of discrimination—for example, community groups who self-identify as being from a Black and Asian Minority Ethnic Group with the following ancestral identities—Caribbean, African,^3^ and Pakistani.^4^

The Tuskegee Syphilis Study^5^ and the story of Henrietta Lacks^6^ are relatively well-known examples cited as contributing to mistrust in medicine and research. Although these are US examples, some UK communities will have a shared history with African American people of slavery and European colonization. This largely includes those who self-identify as being from Black, Asian, and Minority Ethnic Groups. Medical experimentation and lack of informed consent were commonplace in their pre-colonial experiences.^7^^,^^8^ However, such exploitation is not resigned to the distant past. In recent memory of UK communities is the 1996 meningitis outbreak in Nigeria, in which Pfizer carried out unapproved drug testing on children^9^^,^^10^; and, unethical clinical trial practices in India^11^^,^^12^ and the 2020 outrage caused by French doctors Jean-Paul Mira and Camille Locht suggesting that testing new COVID-19 vaccines could be carried out on African populations^13^ constantly reignite fears of science’s exploitative history.^14^ Furthermore, disproportionately poor health outcomes for Black and Asian communities in the UK^15^^,^^16^ may also reinforce beliefs that scientific research does not improve well-being, and may even perpetuate existing inequalities. Mistrust is often linked to generational experiences of discrimination and the systemic racism and social inequity this perpetuates.^17^ For Black, Asian, and Minority Ethnic Groups in the UK, their past and present experiences may understandably play a potential part in fears toward science and genetic research.^18^

People from socio-economically disadvantaged backgrounds tend to be less positive about, and less engaged with, science (although research on attitudes toward genomics is scarce).^19^ Genomic datasets are known to consist of DNA from predominantly White, Northern European populations and there are repeated calls to diversify such databases^20^^,^^21^ and with specific policy calls to recruit participants from missing community groups^3^ and recognition that attention to cultural and linguistic sensitivity is paramount^22^ and yet there is limited evidence of how to operationalize this sensitively at scale, specifically in the UK.

While there is some suggestion that the increased prominence of genomics as discussed in relation to the COVID pandemic (e.g., use of the word “variant”) has contributed to increased self-reported familiarity with genetics,^23^ many questions still surround how to effectively engage public audiences who emotionally detach as soon as they hear something about science,^24^ potentially believing “this isn’t for me,” “I won’t understand it,”^3^ or at worst perceiving that “the science will be used against me.”

Disengagement has practical consequences. Outside the specialist world of genomics, none of us really need to understand the technical details around what a variant actually is, the processes of genome sequencing, and the practicalities of data harnessing. However, as genomics becomes part of everyday healthcare,^3^ some level of familiarity with the applications and implications of genomic science are likely to be needed if publics are required to confidently access the tools of genomic medicine.

As co-authors we have a lived, professional experience of working directly with patient and public audiences and this has taught us that language and its tone play a vital role in determining whether publics feel as though genetics is of relevance to them, whether they feel at ease and confident to discuss it, and whether they feel discussing it can make a difference. We are also aware that there is no universally accepted, nor evidence-based approach for introducing the topic of genomics to audiences who are not specifically seeking out information. When writing a participant information sheet, a patient leaflet, or website text for clinical or research projects we have all used different framings for what genes do and what genomics can offer society. For example, we might frame DNA as *a molecule that contains our unique genetic code*. *Like a recipe book*, *it holds the instruction for making all the proteins in our bodies* and genetic testing as a key to better health: *genetic testing can help anticipate and reduce the risk for certain diseases and conditions before they ever develop*. And, while we assume that we know what public audiences think and feel when they read these texts, we have not seen research before that has intentionally explored an intuitive reaction to these framings and hence our research here aims to do this.

In the work reported here, we explored the views, attitudes, and reactions of members of the public regarding common language used to communicate genomics, with a particular focus on those groups traditionally excluded from, or under-represented in, genomics research. The aims were to understand whether there were any differences between “what we say” and “what people hear” with respect to language around genomics, and what strategies or approaches could be more effective to connect with these audiences.

## Materials and methods

### Study design

The research was commissioned and funded by Wellcome Connecting Science and Genomics England and was designed and delivered by the market research company Maslansky, which specializes in researching and providing evidence for language strategies. The project received a favorable ethical review from the Sanger Institute Connecting Science Research Ethics Committee (Study: 002-22).

We delivered a series of 16 focus groups with 100 participants to test out their responses to typically used phrases and framings of genomics for non-expert public audiences. The focus groups responded to a series of “language stimuli” (i.e., short messages of around 300 words), each stimulus provided a different framing of what genomics is and can offer (see supplemental information for details of the language stimuli). The content for these stimuli was provided by the Genomics England and Wellcome Connecting Science staff (co-authors A.M., A.C., R.M., C.P., S.H., and V.P.)—all experts in writing copy for patient and public audiences on the implications and application of genomic technology. Such staff have worked in different non-profit organizations that deliver genomics research, commit to diversifying genomic datasets, and deliver research that directly impacts on genomic medicine services in the National Health Service in the UK. The content stimuli were based on typical text the staff had previously written for patient information leaflets used in the National Health Service, participant information sheets used in research, website content for non-profit genomics research organizations, and public engagement materials. The rigor of each language stimuli design was cross-checked, edited, and agreed upon between the co-authors. The content stimuli were simplified by the market research linguists and re-written as a script, an actor was filmed reading the script, and the films were shown to the focus group participants. The actor (a Black British woman) was selected to fit with the target audience. The background and clothing were deliberately plain, and the actor was asked to talk in as neutral a tone as possible to minimize influencing participants’ responses.

We piloted language stimuli that explained the potential benefits and value of genomics, covering six different approaches to discussing the topic. These included conversation summarized via:

(1) Collective benefits: it’s good for all of us, e.g., “By studying what makes individuals like you and me our unique selves, scientists can learn more about our health and discover new ways to treat and cure disease for everybody.”
(2) Collective benefits: leveling up the playing field, e.g., “Our genes can be the key to how we ensure we are all provided for, equally and fairly. By comparing your genes with those of other people who share a similar heritage, researchers can spot patterns and learn more about how they affect your health. The result is better healthcare for you and others in your community.”
(3) Personal benefits: it’s good for me, e.g., “By looking at the unique set of quirks and glitches in your DNA, scientists can understand a lot about how to give you the best healthcare.”
(4) Personal benefits: my contribution lives on, e.g., “Your genetic code holds the answers to future medical discoveries.”
(5) Scientific benefits: you can be part of fighting disease, e.g., “Each person who gives their DNA becomes part of a quest for cures and new treatments for diseases like heart disease and diabetes.”
(6) Scientific benefits: it’s key to better health, e.g., “Genetic testing can help anticipate and reduce the risk for certain diseases and disorders before they ever develop.”

We also used six framings of language that aimed to anticipate and address the sense of misunderstandings and mistrust that we knew some public audiences have about genomics, via the following concepts.

(1) Testing is your choice, e.g., “You’re the only one who can decide what’s best for you and your family. And you have the right to all the information you need to make the best choices for you.”
(2) Genetic testing predicts but doesn’t determine your health, e.g., “Our genomes contain all the information needed to build us and allow us to grow and develop. But while your genome can help make predictions, it can’t tell you for sure what will happen in the future.”
(3) Minimizing exceptionality of genetic testing, e.g., “Genetic testing isn’t a replacement for the tools doctors use today, it’s just one more piece of information that works alongside everything else, allowing your doctor to make the best decisions possible for your current and future health.”
(4) Privacy: you have control how your data are used, e.g., “You control whether you share your genetic information with others.”
(5) Privacy: governance and regulations, e.g., “When you take a genetic test for medical reasons, only members of your care team, like your doctor, can access the results. And if you consent for your genetic data to be used for medical research, your identifying information is removed before your data is ever available to use in research.”
(6) Acknowledging the specific concerns of people from Black and Asian Minority Ethnic groups, e.g., “We know that a lot of people have questions—and even concerns—about giving permission for their genes to be used in research. And studies have shown that, in general, concerns among ethnic minorities can be even greater. And there are real reasons for this.”

The first 6 focus groups (n = 34 participants) were conducted and filmed via Zoom in June 2021. Preliminary analysis was completed and an additional 10 Zoom focus groups (n = 66 participants) were then carried out in December 2021 to further test the nuances of the messages and differences between target audiences’ responses with a larger sample. With consent, all focus groups were filmed to capture non-verbal cues that could be used in the interpretation of qualitative data and for dissemination purposes.

### Participants details

Participants were members of a market research company panel based in the UK and were screened based on self-reported data on ethnicity and socio-economic status, including educational attainment (people without a university degree), employment status, and occupation (mapped against lower income levels). Potential participants were invited by the company to take part in an online questionnaire to screen for an upcoming study on an undisclosed topic and received payment for their time. We aimed to recruit four groups of the British public: those self-identifying with three ancestral groups: (1) Black African, (2) Black Caribbean, (3) Pakistani, and (4) a final group of participants of various ethnicities who had lower educational attainment, lower income levels, and occupation categories indicative of socio-economic disadvantage. Eligibility criteria included: (1) socio-demographic details (i.e., being part of one of the four target groups), (2) limited familiarity with genomics, (3) lack of direct experience of genomics through either having a genetic condition in the family or having taken part in genetic testing. The market research company used their own proprietary methods to combine lower educational attainment, lower income levels, and occupation categories into a category of “socio-economic disadvantage.”

Familiarity with genomics was self-reported using a scale from 0 to 5 (0 meaning no familiarity whatsoever, 5 meaning great familiarity). Participants who self-reported limited familiarity with genomics (0–3) were selected for the study. Sampling, consent taking, and recruitment were undertaken by the market research company. All participants provided written consent to take part in the study, to their focus groups being filmed, and for the filmed footage (including a visual image of the participant) to be shared on a publicly available platform such as YouTube so that the results of the study could be disseminated widely (see consent details in supplemental information for details of the consent clauses).

### Data collection and analysis

The filmed focus groups were conducted online using Zoom and each session lasted approximately 2 h. A semi-structured guide was used to explore participants’ views and concerns (if concerns were present) on genomics. Participants were then shown a series of videos of an actor reading selected language stimuli. The language stimuli were randomly selected at first and feedback from the completed sessions was used to refine the selection in the second wave of focus groups.

Using a methodology called Resonance Dial Testing,^25^ participants were invited to use a dial that they could click on, from their keyboard, to capture moment-by-moment reactions to the messages in real time. The 0 to 100 dial was centered at 50 at the start of each video, and participants were encouraged to continuously use the full range of the dial to rate specific passages, words, and analogies based on their gut feeling and immediate reactions. The responses that deviated from the mid-50 point were indicative of a collective group feeling about the linguistic content of the video. These were then used as a prompt to guide the open-ended questions and group discussion that followed, to further explore the reasoning behind participants’ responses. The analysis below focused on qualitative data from the focus group discussion.

The contents of the first wave of focus groups were analyzed by the market research professionals (co-authors K.Y., S.P., T.H., A.C., A.L., J.C., and S.L.) and preliminary insights were shared with the whole team. These original insights were deemed so significant that additional funding was sought by A.M. and V.P. to increase the number of focus groups so that the nuance of the initial research could be explored in more depth. The final, full cohort of 16 focus groups (videos and the anonymized transcripts) were then shared with the Wellcome Connecting Science and Genomics England co-authors who completed the analysis for this article. Here, the academic social science team independently coded the anonymous, written transcripts and analyzed them following established principles of thematic analysis.^26^

Coding was inductive and themes were created using cross-comparison analysis across the whole dataset until saturation was reached. The majority of the coding was done by co-author A.C., with discussion until agreement was reached on codes, analysis and interpretation with co-authors R.M., C.P., and S.H. This form of analysis is data driven, as opposed to driven by specific theoretical frameworks.

## Results

Our sample included 100 adult participants of mixed ages and self-identified gender, English was the first language for all participants. According to the self-identified socio-demographic data provided (including ethnicity) there were 25 people in each of the following groups: (1) participants who self-identified as being from a Black African background, (2) participants who self-identified as being from a Black Caribbean background, (3) participants who self-identified as being from a Pakistani background, and (4) a final group of participants of various ethnicities who had lower educational attainment, lower income levels, and occupation categories indicative of socio-economic disadvantage.

We first present participants’ responses to the language stimuli, and then outline a series of recommendations on optimized communication approaches, based on the data. These consider: (1) the content and structure of the message, (2) specific words and metaphors, and (3) common framing pitfalls to avoid.

### What we say and what they hear: Common approaches to talking about genomics do not resonate

Excerpts of filmed focus groups to demonstrate reactions to language stimuli across all groups (see website for movie/video from focus groups).

As powerfully shown in the above movie, participants expressed clear mistrust and cynicism in response to all the language stimuli that framed genomics through its potential benefits. Starting the conversation with the collective scientific and health benefits very clearly failed to create a connection, in fact, it actively raised concerns (see Table 1).

**Table 1:** Current linguistic approaches and why they do not work

What we currently do	What it involves	Language tested	Why it does not work
Lead with scientific benefits	Fails to articulate personal significance	“Your contribution today could be the key to discoveries that can help future generations. And it could live on to change the world as scientists continue to use it into the future.”	“It was very sciency … We want to be cured now. Not in the future or not when we’re dead.” (Participant from the disadvantaged socio-demographic group)
	Assumes trust that isn’t given	“By looking at the unique set of quirks and glitches in your DNA, scientists can understand a lot about how to give you the best healthcare.”	“Scientists tell us this about COVID, that about COVID. And a lot of the information is ambiguous, and it changes every day …. Just for me, it doesn’t hold any trust and any value.” (Participant from the Black African group)
Lead with health benefits	Fails to acknowledge concerns about historical injustices	“For this research to help everybody, it needs to represent everybody. And that means it needs to include everybody. People from all backgrounds, ethnicities and walks of life. Opting in means more than just saying yes to research. It means saying yes to an equal health care system for all.”	“So, we’ve been used as lab rats, we’ve been used as test dummies. So that’s why we are reluctant.” (Participant self-identifying as from the Black African group) “I heard ‘everybody’; we can all of a sudden help everybody. Okay, so now, what? Are we admitting that there was a point where we weren’t being helped? Even though, let’s talk it as it is, we’ve always known that. We don’t get the same amount of help, or we are not taken seriously. Or, they just ‘okay, it’s sickle cell, they’re black.’” (Participant self-identifying as from the Black Caribbean group)
	Fails to acknowledge concerns about present day inequalities	“The result is better healthcare for you and others in your community. That means improvements like diagnosing diseases earlier and more accurately, finding better, more personalized treatments, and, ultimately, making sure you get the medical care you deserve.”	“There’s a thing called the postcode lottery … It is personalized care because it’s at a price. So yes, maybe you can have that type of treatment; however, if it’s a cost, where we are within the budget that we have, you might not get it.” (Participant from the disadvantaged socio-demographic group) “If you look at auto-immune disorders that affect black people, they’re never looked at. And black women, they’re most likely to die in childbirth so they’re always overlooked. So now, they’re going to help us, absolute they won’t. That won’t happen.” (Participant self-identifying as from the Black Caribbean group)
	Oversimplifies the message	“In your DNA scientists can understand a lot about how to give you the best healthcare”	“I don’t even know if scientists understand DNA fully. From what I know, scientists only know a very small amount about our DNA and a lot of our DNA is not comprehendible at the moment. So, I think there’s a lot more to be found through science.” (Participant from the disadvantaged socio-demographic group) “A lot of the factors that contribute to our healthcare, perhaps, are driven by the fact that we are, generally, in the UK, less socio-economically well-off than other ethnicities or other racial groups. It doesn’t necessarily mean that us providing our data will necessarily provide better healthcare.” (Participant self-identifying as from the Pakistani group)
	Assumes trust that isn’t given	“Using genetic testing, doctors and scientists can effectively Google the wealth of information that’s stored in your DNA … If you choose to have a genetic test, scientists along with several government agencies and non-profit organizations are standing by to answer your questions and make sure you have everything you need to make the right decision for you.”	“I would never, because you just don’t know what they’re really using your information for. Can it be used against you later on? What they’re developing with it? They don’t really communicate things well, in general anyway. So, any type of government access for me would be a no, like I just, no.” (Participant self-identifying as from the Black African group)

Participants expressed mistrust in science (“We’ve been used like lab rats”). Many cited examples of racism in science, including historical injustices (e.g., the Henrietta Lacks case) as well as present-day issues (e.g., lack of diversity in clinical trials and the unequal funding for research on diseases with a higher prevalence among particular groups). Others, including participants self-identifying as from a Pakistani background, mentioned general weariness among older generations about feeling disengaged from science. For some, the association with science evoked high-profile and controversial applications of genomics, such as cloning, or gene editing, and even dystopian “sci-fi” imaginaries. Some participants of color expressed the view that science cannot be trusted to be reliable and gave COVID-19 as an example of where advice on how to manage the pandemic changed and could appear contradictory. Even when not overtly critical of science, participants’ responses indicated a general disengagement from it. For a few participants, genomics was something they learnt at school as part of formal scientific education, so it had an intuitive connection with science (which they felt disengaged from). When asked to elaborate on these associations, however, participants tended to give short and generic answers, suggesting that this aspect of genomics failed to resonate in a way that was personally significant to them.

Compared with generic scientific benefits, which could feel distant and abstract, participants spontaneously identified a range of potential health benefits that could have a tangible impact on their lives. Despite this, they also questioned “*who* benefits,” i.e., who will benefit the most, and who will be left behind? Participants were particularly suspicious of claims suggesting genomics would benefit “everybody” and considered that people like them would be more likely to be excluded.

These responses were informed by a range of views and personal experiences. Participants cited examples of how medical research had failed their communities or exploited them for someone else’s benefit. Participants also mentioned the effects of systemic racism on access to, and quality of, health care. Finally, participants from all groups mentioned the intersectionality of racism with other forms of inequalities, particularly socio-economic inequalities, and the implications for equity of access to healthcare as a reason why they feared they would likely miss out on potential health benefits.

A second reason behind participants’ skepticism was the lack of trust in those who should deliver such benefits. Participants were particularly mistrustful of private companies and the government, both assumed to have “ulterior motives.” Scientists were unlikely to be considered trustworthy for the reasons cited above. Some participants also voiced distrust for the National Health Service and for regulating bodies, neither of which was considered capable of protecting against data breaches. While the reasons behind the lack of trust in each of these actors might be different, the effect was similar in that it appeared to be associated with participants’ suspicion of declared intentions, including claims of potential health benefits from genomic research.

Finally, some participants found the promised benefits “too good to be true.” Specifically, they cited the limitations of current knowledge of the genome, the long and uncertain process to translate scientific discoveries into clinical benefits, the lack of resources presumably needed to deliver genomic/personalized medicine, and the importance of wider determinants of health. As a result, messages that appeared to over-promise or over-simplify potential benefits were perceived as disingenuous. Some participants compared this type of language with a “sale pitch” filled with “buzzwords.” Others described it as “manipulative,” “coercive,” and “deceitful.”

### What could work instead: Meet the audience where they are at

#### Content and structure of the message

Because of the issues discussed above, messages leading with the potential benefits of genomics are, at best, likely to fall flat, and at worst they could raise suspicion. It thus appears important not to begin the conversation with an explanation of the benefits of genomics (these come later) but to initiate “the hello” by meeting people where they are conversationally and addressing what is important to them first (see Table 2).

**Table 2:** Content and structure of the optimized message

What we can do	What it involves	Language tested	Why it works
Acknowledge doubts and distrust	Validate concerns about past and present injustices	“We know that a lot of people have questions—and even concerns—about giving permission for their genes to be used in research. And studies have shown that, in general, concerns among ethnic minorities can be even greater. And there are real reasons for this. Some are connected to personal experiences and some to historical injustice. These concerns are real.”	“It makes me feel a bit more seen, that they can understand and that they have some appreciation for other points of view … Especially in contrast to [*the previousmessages*], where it was almost good to get as many people to donate, it is a lot more nuanced [*this updatedmessage*] that still outlines the benefits.” (Participant self-identifying as from the Black African group) “Acknowledgment that ethnic minority people are very skeptical, acknowledgment that in the past history, that this particular client group has been not treated fairly in regards of treatment for health wise. So, I think acknowledging that, I think that has been positive. So not ignoring the past. (Participant self-identifying as from the Black Caribbean group)
	Validate doubts due to a lack of familiarity with genomics	“If you’re wondering what genetic testing is all about, you’re not alone. Healthcare can be confusing enough as it is, and it would be surprising if people didn’t have questions about things like DNA testing”	“You can resonate with it because you know, when you asked us at the start all these questions, a lot of us, we really didn’t know anything about it. So it makes you feel more accepted by the fact that you have kind of no knowledge or not very broad knowledge on the subject.” (Participant from the disadvantaged socio-demographic group)
Empower people	Frame it as a voluntary choice	“It is your health, your genes, and your choice.”	“I think it is right. It is my choice. It is almost like there is no pressure, really.” (Participant self-identifying as from the Black African group)
	Emphasize control where relevant	“The most important thing to know is this: you control whether you share your genetic information with others. One reason to do so is to receive better healthcare. In this case, the results of a genetic test will remain private, just like your other medical records, and only you and your doctor will have access. Another reason is to volunteer to have your genetic information used in research for new treatments. In this case, your genetic information and your medical history is protected in a secure database.”	“I think it’s good that you have a right over it, so it’s not just like they just take it and then you give it to them and they can do whatever they want. You still have your say in it.” (Participant from the disadvantaged socio-demographic group)
Calm concerns	Avoid emphasizing novelty. Instead, ground genomics in what is familiar	“Today, your doctor can use a simple blood test to uncover a universe of information, like your red blood cell count or your cholesterol. In much the same way, they can look at your genes to get a more complete picture of your health, like your risk for developing certain conditions. Ultimately this extra information means they can make better decisions for you and with you. Genetic testing isn’t a replacement for the tools doctors use today, it’s just one more piece of information that works alongside everything else”	“I thought it was interesting. The part where it said it might be unfamiliar for you to hear about it, it’s just like other tests and the reassurance it’s giving you the best care possible, I think that was quite reassuring and also the fact that it was just saying we just want to improve ways in trying to make your health better.” (Participant self-identifying as from the Black Caribbean group)
	Build some context around it: be specific about what is and is not involved	“When you put your genetic information into the care of a doctor or a researcher, it still belongs to you and you have a say in how it’s used. That right is protected by several different laws that were created to make sure your data is handled securely and responsibly … The main way the privacy of your data is protected is by restricting who can see it. When you take a genetic test for medical reasons, only members of your care team—like your doctor—can access the results.”	“In a way it was very reassuring, and I think it was the first video that actually talked mostly about the security and mostly about not to get you in some way, but to reassure you and protect your information, and I wish it was something like this from the beginning. I would feel much more reassured and much more secure.” (Participant from the disadvantaged socio-demographic group) “I think that’s one of the questions I probably had in my head, to be honest, throughout this session was whether our identity would be anonymized when it’s going to researchers. So, that’s quite good to know.” (Participant self-identifying as from the Pakistani group)
Introduce benefits after the preparatory work above has been done	Move to personal benefits by articulating the specific relevance for the target audience	“It’s important you get the information you need on why you might—or might not—want to consider opting in to sharing your genetic information to help create better, fairer, and more personalized medicine for you and your family, and families like yours.” “When scientists are able to compare more people from many different backgrounds, they can gather more insights and, over time, help more people.”	“It did mention family a bit and that sounded more like immediate family. I can resonate with that quite deeply to be fair. It’s a bit like it would benefit me, benefit my immediate family, my loved ones around me, and then the future.” (Participant from the disadvantaged socio-demographic group) “People like that, who aren’t rich, middle-class: are they also going to benefit from this? Because at the end of the day, that’s where I came from, to where I am now, and I would like for my import not to be disregarded just because of race or what your class is … So, me helping gather DNA, I would like people from my roots to also benefit from this.” (Participant self-identifying as from the Pakistani group)
	State concrete, tangible benefits and (where possible) incorporate specific examples	“In the near future, we’ll be able to use a bit of blood or saliva to do a DNA test. The results of this test can mean less guesswork and the ability to diagnose diseases like breast cancer earlier and more accurately. And the more people who share their DNA for researchers to study, the more medicine can be precisely designed to work for you and your family.”	“I like it. I thought it was the strongest out of the ones that we’ve seen so far. They try to appeal to you as an individual. They explained what it was going to be used for. They also dropped in the C word, which most people know somebody or has some kind of connection with that. That kind of makes like your ears prick up more and you pay attention.” (Participant self-identifying as from the Black African group)
	Finally, nod at the bigger picture by appealing to wider, collective benefits	“If you do participate, your doctor can spot patterns and learn more about how they affect your health by comparing your genes with people who share a similar heritage. The result is better healthcare for you and others in your community.”	“Well, probably it was not more motivating for me personally because obviously I’m not a minority ethnic group, although my husband is. Not for me, but it gave me a better feeling about the whole project, maybe. Yes. I’d say it does make me feel a bit more positive about the whole concept of it.” (Participant from the disadvantaged socio-demographic group)

#### Words and metaphors

Words and metaphors conveyed different feelings to our participants. This is not to say that certain words are better or worse than others, but that they tend to carry specific nuances that could indicate they are only appropriate in certain contexts and with certain audiences.

#### Genome/DNA/gene

*Gene* and *DNA* were both familiar words among our participants. *Genes* were closely associated with concepts of identity, family, and heritage (“it’s in your genes”), whereas *DNA* had a more scientific connotation; depending on the context, it could be perceived as more accurate but could also turn off our audience *Genome* was perceived as overly technical and obscure.

“I don't really know what it [genome] means, so I'll say this doesn't speak to me.”

(Participant self-identifying as from the Pakistani group)

“When I hear the word genes, I find myself looking at, like say, my grandparents, my grandchildren, my daughter. I look at that aspect of it. Almost like they’re naturally there, if you get my point. Because I think that genes are not just necessarily blood cells, I think it’s character, person being, whereas with DNA, when I think of DNA, [laughs], it always takes me to the crime element, it takes me to evidence and things like that. And so, if I’m looking at scientists and governments and all of that, I think that if they get hold of our DNA, what could they actually do with it?”

(Participant self-identifying as from the Black Caribbean group)

#### Gifting/sharing/allowing/opting in

When describing taking part in research, “passive” language (“allowing”) was the least preferred option. “Gifting” and “sharing” were appreciated because they emphasized active choice; however, these terms were also interpreted as meaning that there was an ability to lose control over data after it has been shared. Gifting was also perceived as “intimate” and could be associated with deceased donation (e.g., of organs). “Opting in/out” was the most effective option because it implied the decision could be revoked.

“When you pass over this information, who owns it? So, you’re sharing it—are you giving it away forever?”

(Participant self-identifying as from the Black African group)

“When I hear gift, it means to gift it with no questions to be fair, you know it’s a gift, you can use it how you like to use it there’s going to be no questions asked about it.”

(Participant self-identifying as from the Pakistani group)

“I just think there is a certain connotation with gifting, like I am giving it to you like a present. It is a very serious study. Someone who … gives their lungs for scientific study isn’t gifting it, it is usually because they die.”

(Participant self-identifying as from the Black African group)

“I just think that bit [*opting in to research*] gives you the option if it’s something you want to do. It’s not something that’s forced upon you, and you have to do it. So, I think it’s just the option that you can do it, if you want to, which speaks to me more than ‘allowing us to study’.”

(Participant from the disadvantaged socio-economic group)

#### Glitches/variations/similarities and differences

Overall, participants indicated a preference for plain and neutral language to discuss genetic variation. The phrasing “variations in genes” was the preferred option. “Similarities and differences” was also rated positively, although leaning too much on the differences could be perceived as “dividing.” The metaphor of “glitches” was considered by some as too scientific and cold.

“The word glitches really make it more scientific. When you think of glitches, you think of like a robot or something that has failed.”

(Participant self-identifying as from the Black African group)

“I think the variation bit is a softer, better, more professional term.”

(Participant self-identifying as from the Pakistani group)

“[Similarities and differences] sound more like you're actually being honest about what's going on.”

(Participant self-identifying as from the Black African group)

#### Personalized/precise/tailor-made

Overall, participants were familiar with the concept of personalized medicine and felt positive about it. “Personalized” was appealing because it spoke to an individualized, patient-centered approach. However, it could also evoke ideas of private, and therefore, expensive (out of reach) care. The terms “precise” and “tailor-made” were appreciated because they are self-explanatory and signaled efficacy and accuracy.

“[Personalized] would be more accurate towards your personal needs.”

(Participant from the disadvantaged socio-economic group)

“I think ‘precisely designed’ really does go a lot to the roots of being tailored for me rather than just personalized because personalized is like a credit card that's personalized for a group of people.”

(Participant self-identifying as from the Pakistani group)

“It says it’s going to be more precise, it’s going to be personalized. Now when you talk about personalized, it’s individual. So, what you are actually trying to say or sell? Can you have five people in a family all having a different package, and how much of this is going to cost us?"

(Participant self-identifying as from the Black Caribbean group)

### Common framing pitfalls to avoid

Finally, participants’ responses highlighted some common pitfalls that, if not avoided, could invalidate the overall message.

#### Genuine inclusivity requires attention to categories and context

Participants responded positively to messages tailor-made for them. For example, participants from self-identified Black African and Caribbean ancestral backgrounds expressed a preference for specific language (e.g., Black), as opposed to more generic wording (e.g., “ethnic minorities”). If not appropriately framed, however, zooming in on a particular group could also make people feel “targeted,” as opposed to “included.” Particularly when addressing communities that have been traditionally marginalized, this approach can have a patronizing or stigmatizing connotation.

“But in the whole society, we’re regarded as a minority, so I think we should just say what we are, or it should just be said what we are, because even if you go back to Black Lives Matter, why are we so uncomfortable with using the terminology Black? Maybe had they said Ethnic Minorities Matter maybe they wouldn’t have felt so bad about it? Do you understand the point I’m trying to make? At the end of the day, if we’re trying to address people of color, or if you’re trying to address Asian, if you’re trying to address Black, just say it.”

(Participant self-identifying as from the Black Caribbean group)

“I do believe that when they are trying to encourage people from certain communities to donate, that you can go about that in a different way. But I think it feels like I'm being side-lined in … And I consider myself British, but it still feels like I'm being side-lined all of a sudden.”

(Participant from the disadvantaged socio-economic group)

It is also important to consider that the categories used, including by participants themselves, are neither fixed nor mutually exclusive, and can change depending on context.

#### Vagueness invites skepticism

The more distant and abstract a new technology appears, the more it appeared to raise fears and suspicions. When the language was lacking concrete details, space was easily created for skepticism:

“What I think may have improved it [*the language stimuli*] is to understand how getting anything from me physically translates into better healthcare. It sounded like a great sales pitch, but it didn't really tell me how we would get from Point A to Point B.”

(Participant self-identifying as from the Black African group)

*Language tested:* “For this research to help everybody, it needs to represent everybody. And that means it needs to include everybody.”

*Response:* “This one , to me … It's quite negative, had a negative impact. I just actually stopped listening to that. At one point it was just to me, emotional blackmail. This sort of utilitarian "for the greater good, for everybody."

(Participant self-identifying as from the Black African group)

What type of information might be relevant depends on the specific context; however, issues highlighted by participants included: how the data will be used; who is involved; what regulations and governance will be in place; what the project does *not* involve (e.g., cloning).

#### Do not amplify concerns

Addressing participants’ concerns appears to be important but may unintentionally amplify fear. Language that evoked danger and cited examples of how things could go wrong was more likely to heighten rather than assuage concerns.

*Language tested:* “Few pieces of data are as precious and personal as your genetic information—so it’s critically important that you’re in control of how it’s used.”

*Response:* “It left me a bit skeptical of it. It’s tricky because they’ve mentioned it and it’s made me skeptical of it!”

(Participant from the disadvantaged socio-economic group)

Instead, an approach that gave people *permission* to be concerned was more likely to be experienced as validating.

*Language tested:* “If you're wondering what genetic testing is all about, you're not alone. Healthcare can be confusing enough as it is, and it would be surprising if people didn't have questions about things like DNA testing.”

*Response:* “It makes you feel more accepted by the fact that you have kind of no knowledge or not very broad knowledge on the subject.”

(Participant from the disadvantaged socio-economic group)

#### “Choice” can be easily misinterpreted

Participants valued a language that emphasized choice and put them in control. These concepts, however, can be loaded with pre-existing associations, particularly in a healthcare context. In a minority of cases, the *choice to take part* in genomic research was confused with the idea of *making informed health choices* based on the results of genomic testing. Clarity on this point is key to avoid over-simplifying potential benefits.

## Discussion

Our research has demonstrated that everyday talk about genomics currently used by researchers and clinicians alike has the potential to alienate already disengaged public audiences. We interpret our findings not in terms of illiteracy about genomics but as illustrative of the very real socio-historical inequities and inequalities that exist for people from marginalized communities. The conversations about genetics that led with the science and its benefits were triggering for participants, and this revealed itself as cynicism and mistrust. Thus, continuing to frame our science only through its benefits, however well-meaning, has the risk of doing harm. The genomics community has an obligation to take heed of the voices represented in this work—not only is this ethically just (and at a minimum, courteous) to care about how one’s language lands with the target audience but it is also pivotal if the genomics industry wants to embark on conversations with community groups about including them in genetic research, thus diversifying the ancestral and ethnic background of existing datasets.

Clinicians and researchers working in the field of genomics are very familiar with “genomic talk” about the implications of the technology and the applications for society; however, one-way dissemination models of communication allow scant time for any consideration on how this talk is received and whether it has the desired impact, let alone whether it may even be doing more harm than good. Information about genomics is delivered via non-profit, for-profit organizations, clinicians, researchers, teachers, educators, the media, and often replicates the same framings and linguistic patterns that surround the industry—i.e., by leading with the science and explaining the health benefits. However, as our research has shown, we must not be complacent in our acceptance that this works for everyone—we have touched the surface in showing that it certainly does not work for the community groups we have interviewed here. It may not even work for many other public audiences and thus more research is needed to actually test the linguistic framings that are routinely used.

Stakeholder and public engagement are “widely lauded as an important methodology for improving clinical, scientific, and public health policy decision-making.”^27^ Without active and continued public engagement in the field of genomics, its potential to protect public and population health will remain unfulfilled.^20^^,^^28^ Furthermore, as genomic medicine becomes increasingly available, asymmetric uptake may serve to further increase health inequalities.

The UK has a vibrant genomics ecosystem and much written material is already in place that describes what genomics is and why it is relevant to us all. However, there is no universally accepted, evidence-based framings for language around genomics that are known to work, particularly for audiences who have already expressed a mistrust and disconnection to the science.^29^ A step change is needed to reach public audiences, where they are at, if the information around genomics is to radiate across the whole of society. In common with wider science engagement programs, genomics engagement has often largely relied on the audience already having some level of interest in, connection to, or knowledge of genomics, something that often relies on high prior “science capital.”^30^ As a result, existing engagement strategies are hindered from fostering broad public awareness and trust in genomics because, by their very nature, they will never reach audiences who are currently very disengaged and may, in fact, exclude them.^31^

We have shown that paying close attention to “the hello” is important for engaging with disengaged community groups. The order of the conversation matters and leading with the benefits of genomics may not be appropriate for already disengaged audiences; we found this was greeted with suspicion and cynicism from our participants. Instead, we have shown the importance of preparatory conversation which acknowledges science and genetics have a difficult history for communities that have felt marginalized. We have also shown that participants value the recognition that a lack of familiarity with genomics is common and that participation in any genetic research is always voluntary. This *easing into the* conversation appears potentially significant in helping the engagement encounter and more research is needed to understand at what point publics are then ready to embrace the details of the science. We have also shown that discussions about genomics need to be specific, with a clear articulation of the tangible personal benefits, using examples, as well as details about the collective benefits of the science.

Those who are actively disengaged are the “hardest to reach” public group for science engagement activities. They may also be most likely to resist as they have no prior desire to engage. However, if some familiarity with genomics is necessary to realize its clinical and public health benefits, failure to connect with such disconnected audiences may significantly limit the possibilities for the field and the promised benefits for society.

How we collectively talk about genomics and the language we use is thus of vital importance to avoid alienating the very people our science exists to serve. This is particularly important for actors working in the genomics sphere (whether clinical, research, non-profit or for-profit) who “talk about genomics” with public audiences. This does not necessarily mean promoting or proselytizing the promise of genomics but recognizing that “genomic talk” intersects with people’s existing cultural meanings, references, and historical connections, and their hopes and fears related to science and medicine.^32^ In turn, this means recognizing that there are many ways of seeing and knowing genetics and this puts an importance on the emotional as well as cognitive content of communication.^33^

Scientific racism is an upstream sociological causal factor and a historical fact. And, as demonstrated in our findings, this is an issue that participants articulated directly in response to all of our linguistic framings that led with the benefits of genetics. We neither prompted them for this, nor sought it out, but nevertheless it was clearly expressed. The enthusiasm of scientists and clinicians to extoll the benefits of genomics, however well intentioned, should be given thoughtful consideration. As our research demonstrates, what we say and what people hear can be worlds apart. Ignorance of the intense emotion about present-day scientific racism is no defense and it is insufficient to claim we are neutral actors—“I am simply explaining the benefits of the science!”—when the impact of the genetics research is open to subjective interpretation, positionality, intense debate, and sits within the context of misuse.^18^

For those involved in the development of genomic medicine, acknowledging this, and working toward a shared approach may mitigate the risk of a negative public response with the potential to derail progress in the implementation of genomics,^34^ while also allowing us to rethink how and why we communicate around genomics. It is vital that all of us working in genomics take responsibility for identifying, creating, sharing, and working within evidence-based communications strategies that are meaningful for the populations we purport to serve.^29^

### Limitations of the study

The recruitment criteria (ethnicity and socio-economic characteristics) are not homogeneous. The categories used are not necessarily experienced in the same way by participants (ethnicity may be visible, socioeconomic status is not necessarily so). These differences could shape how participants understood their own positionality in the study (e.g., as a Black member of the public vs. as a generic member of the public), and the personal implications of the topic. Further research is therefore needed to explore similarities, differences, and intersectionality between different audiences. We also do not know if the ethnicity of the video narrator may have biased or impacted participants’ perceptions. There is also the possibility of bias from the involvement of the market research company in that the audience they recruit for their panels is possibly more used to working with advertising research as opposed to academic research (although the market research company themselves reassured us that their public panels are familiar in working with both). Focus group research can also be tainted by Social Desirability Bias^35^ that comes from a “group think” and a need to give answers that are socially acceptable; we attempted to minimize this by working with trained facilitators who were familiar with setting the scene to allow participants to answer honestly and authentically.

## Data and code availability

This study did not generate/analyze code. This published article includes data (both video and written transcripts) generated and analyzed during this study.

## Web resources

Focus groups, https://www.youtube.com/watch?v=TwvoRss1WGA

## Supplemental information

Document S1. Supplemental material

Document S2. Article plus supplemental information
