# Supplementary material for: The legacy of language: What we say, and what people hear, when we talk about genomics
Source: HGG Adv. 2023 Aug 31;4(4):100231. doi: 10.1016/j.xhgg.2023.100231 (PMC10589723; doi:10.1016/j.xhgg.2023.100231)
Supplement: Document S1. Supplemental material [file mmc1.pdf]

HGGA, Volume *4*

Supplemental information

The legacy of language: What we say, and what people hear, when we talk about genomics

Anna Middleton, Alessia Costa, Richard Milne, Christine Patch, Lauren Robarts, Ben Tomlin, Mark Danson, Sasha Henriques, Jerome Atutornu, Ugbaad Aidid, Daniela Boraschi, Catherine Galloway, Keith Yazmir, Sachi Pettit, Tegan Harcourt, Alannah Connolly, Amanda Li, Jacob Cala, Shelby Lake, Julian Borra, and Vivienne Parry

Supplemental Material

This document contains details of:

1. Language stimuli tested
2. Study procedures and ethical issues
3. Consent form for participants

1. Language stimuli tested

Articulating benefits and value of genomics (Why is genomics a good thing?)
Collective benefits: It’s good for all us
Your genes can help guide the way in our search for better health for all. The secret is in our precious DNA, the microscopic code of life. The magic lies in genomics, the field of science that explores and helps us understand the tiny differences between us. For example, there are people who exercise their whole lives and eat a healthy diet, but die of a heart attack at 40. And then there are people who never exercise and eat unhealthy food, but live to be 100. By studying what makes individuals like you and me our unique selves, scientists can learn more about our health and discover new ways to treat and cure disease for everybody. The more of us who feel okay about allowing our DNA to be studied, the better healthcare will be for everyone. When scientists are able to compare more people from many different backgrounds, they can gather more insights and over time help more people. Answers to better health, much like the genome itself, lie within all of us.

Collective benefits: Levelling up the playing field
You can help level the playing field for health and well-being. While everyone deserves the best healthcare, we don't always get treated the same. Sometimes it's about where you live or how much money you have, but it can also be about whether medical research has focused enough on people from a similar background as you. Our genes can be the key to how we ensure we are all provided for, equally and fairly. By comparing your genes with those of other people who share a similar heritage, researchers can spot patterns and learn more about how they affect your health. The result is better healthcare for you and others in your community. That means improvements like diagnosing diseases earlier and more accurately, finding better, more personalised treatments and ultimately, making sure you get the medical care you deserve. For this research to help everybody, it needs to represent everybody. And that means it needs to include everybody - people from all backgrounds, ethnicities, and walks of life. Opting in means more than just saying yes to research. It means saying yes to an equal healthcare system for all.

Personal benefits: It’s good for me
Better medical care might just lie within you. That means better medicine, better ways to figure out what's wrong, and better ways to find the right treatment. And it's all possible thanks to the special chemical code you carry inside. About 99.8% of your DNA is the same as other people, but the 0.2% that's different is what makes you, you. By looking at the unique set of quirks and glitches in your DNA, scientists can understand a lot about how to give you the best healthcare. In the near future, we'll be able to use a bit of blood or saliva to do a DNA test. The results of this

test can mean less guesswork and the ability to diagnose diseases, like breast cancer, earlier and more accurately. And the more people who share their DNA for researchers to study, the more medicine can be precisely designed to work for you and your family. Your DNA does more than just make you unique. It can help you and your family get healthcare that's more precise, personalised, and just better.

Personal benefits: My contribution lives on

Your genetic code holds the answers to future medical discoveries. That's because your DNA is more than just a record of where you came from, or instructions for how you grow and develop. It's how you pass on a piece of yourself for generations to come and how you can leave your mark on medical research now, and into the future. Genetics research has already changed the way we diagnose and treat diseases like cancer, diabetes, and heart disease, but we're just at the beginning of what's possible. The more genetic data we look at, the more we learn. And the more we learn, the closer we get to treatments that get to the root problems of sickness and disease. Your contribution today could be the key to discoveries that can help future generations. And it could live on to change the world as scientists continue to use it into the future. So, when you think about what kind of mark you leave on this world, remember the smallest thing you have to offer can actually leave a big legacy.

Scientific benefits: You can be part of fighting disease

It's easier than we think to save a life. Every day, we each do our bit to make things a little better for those around us – in the last year more so than ever. From helping out a neighbour to donating food or time, doing our part just feels good. It costs us little, and the world is better for it. Now, we can all do our bit to improve healthcare, too. When people are having NHS care, they will be asked if they want to help others by gifting their DNA for research. More and more people are weighing this up. When millions and millions of different people donate their DNA, scientists can learn more about how to stop life-threatening diseases and crippling conditions. Each person who gives their DNA becomes part of a quest for cures and new treatments for diseases like heart disease and diabetes. Becoming a DNA donor is about more than just donating your DNA – it's helping with critically important research that could give someone a second shot at life.

Scientific benefits: It's key to better health

Britons can shape the future of medical care with our DNA, and we're doing it by unlocking the clues and signs inside the tiny bits of data that make us all who we are, our genes. In fact, the NHS is on track to become the world's first health system to look at our genes as part of routine healthcare. This means we are on track to be among the first worldwide to benefit from these advancements. DNA is a molecule that contains our unique genetic code. Like a recipe book, it holds the instruction for making all the proteins in our bodies. When we share the DNA we were born with, doctors can uncover information they can use to provide better care for both ourselves and others in the future. For example, genetic testing can help anticipate and reduce the risk for certain diseases and disorders before they ever develop. Together, we have the potential to make this vision a reality for all people who live in the UK and in the process, we can help millions accelerate research for cures and transform healthcare for the world.

Assuaging fears and concerns (Why shouldn’t I be worried about genomics?)
Testing is your choice
If you're wondering what genetic testing is all about, you're not alone. Healthcare can be confusing enough as it is, and it would be surprising if people didn't have questions about things like DNA testing. Using genetic testing, doctors and scientists can effectively Google the wealth of information that's stored in your DNA. That means they can access unique information about your health that helps them answer important questions like your risk for certain disorders or inherited conditions, or what kinds of treatments might work best for you. If you choose to have a genetic test, scientists along with several government agencies and non-profit organisations are standing by to answer your questions and make sure you have everything you need to make the right decision for you. At the end of the day, you're the only one who can decide what's best for you and your family. And you have the right to all the information you need to make the best choices for you.

Genetic testing predicts but doesn’t determine your health
Our DNA can tell us a lot, but it can’t tell us everything. Studying it can get us a step closer to some answers, but it’s not a crystal ball. In fact, our DNA is just one of many things that affects who we are and who we’ll become. Each of us has a unique combination of DNA, called our genome. Our genomes contain all of the information needed to build us and allow us to grow and develop. But while your genome can help make predictions, it can’t tell you for sure what will happen in the future. And there are lots of things it can’t tell you at all – like what your personality is, who you’ll fall in love with, or even the exact colour of your skin. Studying our genomes can be a powerful way to help us make better decisions about our health. But the information we learn can only help us predict – not determine – the future.

Minimising exceptionality of genetic testing
DNA tests might seem like science fiction, but they're actually quite unremarkable. You might've been anxious about having your first blood test, but now it likely feels like a routine part of any doctor's visit. The same can be said for genetic testing. While it might be unfamiliar when you first heard about it, it's actually just like other tests doctors use to give you the best care possible. Today, your doctor can use a simple blood test to uncover a universe of information, like your red blood cell count or your cholesterol. In much the same way your genes give a more complete picture of your health, like your risk for developing certain conditions. Ultimately this extra information means they can make better decisions for you and with you. Genetic testing isn't a replacement for the tools doctors use today, it's just one more piece of information that works alongside everything else, allowing your doctor to make the best decisions possible for your current and future health.

Privacy: You have control over how your data is used
In our connected world, we share scores of data about ourselves every day, but few pieces of data are as precious and personal as your genetic information, so it's critically important that you're in control of how it's used. The most important thing to know is this. You control whether you share your genetic information with others. One reason to do so is to receive better healthcare. In this case, the results of a genetic test will remain private, just like your other medical records and only you and your doctor will have access. Another reason is to volunteer, to have your genetic information used in research for new treatments. In this case, your genetic information and your medical history is protected in a secure database. Scientists from universities and pharmaceutical

companies, then request to use this information for research into medicine that help people live healthier lives. If you ever decide you don't want more information to be used anymore, you have the right to request that it be erased. When thinking about whether to share your genetic information, just remember, it's your health, it's your genome, it's your choice.

Privacy: Governance and regulations
For as much personal data as we share every day, securing it may not always be our top priority, but when you make the important decision to share your genetic data for research purposes, you deserve to know about all the legal protections in place to secure it. When you share your genetic information with a doctor or a researcher, it still belongs to you. And you have a say in how it's used. It is protected by several different laws that were created to make sure your data is handled securely and responsibly. And these laws insist on the best security, using systems more secure than at most banks. The main way the privacy of your data is protected is by restricting who can see it. When you take a genetic test for medical reasons, only members of your care team, like your doctor, can access the results. And if you consent for your genetic data to be used for medical research, your identifying information is removed before your data is ever available to use in research.

Acknowledging the concerns of Black and Asian Minority Ethnic Groups
We know that a lot of people have questions –and even concerns –about giving permission for their genes to be used in research. And studies have shown that, in general, concerns among ethnic minorities can be even greater. And there are real reasons for this. Some are connected to personal experiences and some to historical injustice. These concerns are real. It's important you get the information you need on why you might –or might not – want to consider opting in to sharing your genetic information to help create better, fairer, and more personalised medicine for you and your family, and families like yours. Understanding our genetics can mean better understanding our health. But the first thing to know about genetic research is that it's up to you whether to participate. It's your health, your genes, and your choice. If you do participate, your doctor can spot patterns and learn more about how they affect your health by comparing your genes with people who share a similar heritage. The result is better healthcare for you and others in your community. That means improvements like diagnosing diseases earlier and more accurately, finding better, more precise treatments, and, ultimately, making sure you get the medical care you deserve. This is one way of making healthcare more fair for you.

2. Study procedures and ethical issues

The project has received a favourable ethical review from the Sanger Institute Connecting Science Research Ethics Committee Study: 002-22. The research was commissioned and funded by Wellcome Connecting Science and Genomics England and was designed and delivered by Maslansky.

Maslansky subcontracted the Schlesinger Group market research company who did the consenting, recruiting and delivered the focus groups. Schesinger is a member of the UK Market Research Society- a voluntary regulatory body that provides governance for the ethical practice of academics working in market research. The industry standard for global social sciences public attitudes

research delivered by market research companies is available at: https://www.schlesingergroup.com/en/company/purpose/.

Wellcome Connecting Science and Genomics England accept that the governance processes are robust enough within the way Schlesinger operates to feel confident that the consent conversation with potential participants was appropriate and meets academic standards. Under GDPR, Schesinger acted as the data controller for the recruitment and conduct of the study. The researchers at Wellcome Connecting Science had no direct contact with any of the research participants and cannot identify anyone to withdraw them from the study and/or film. However, even though participants have given their consent for their visual image to be shared publicly and are aware that by virtue of this, it is difficult to be able to withdraw, we do make the offer of blurring their image. If after seeing the outputs from the research, they change their mind about their visual image continuing to be publicly available, they can contact Wellcome Connecting Science and we will retract the publicly available films that we hold of participants who identify themselves to us.

3. Consent form for participants

- I understand that a video recording of the zoom focus group will be made, sent and stored by our academic partners at Wellcome Connecting Science, Wellcome Genome Campus, Cambridge, UK.
- I understand that clips and written transcripts from my video will be used to demonstrate particular points I make, these will be publicly available for anyone to see on a public facing website or academic presentation.
- I understand that video containing my visual image, but not my name or any other identifying information, will be stored and uploaded onto a video sharing platform such as YouTube or Vimeo, and thus be publicly available for anyone around the world to view.
- I understand that, due to the video recordings and written transcripts being publicly available for viewing, I understand that they may be downloaded by people unconnected to the research and so it is not possible to withdraw or permanently delete them.
- The recordings and written transcripts of the full sessions, which will not include names or other identifying information, will be used in academic presentations, as multimedia attachments to academic papers, and used by both Wellcome and Genomics England in presentations that may be recorded. The main audiences will be academic and related groups, charities, think tanks and commercial audiences in the context of genomics education and awareness raising.
